# Enabling Intelligent Recovery of Critical Materials from Li-Ion Battery through Direct Recycling Process with Internet-of-Things

**DOI:** 10.3390/ma14237153

**Published:** 2021-11-24

**Authors:** Yingqi Lu, Xu Han, Zheng Li

**Affiliations:** 1 Department of Mechanical Engineering, Virginia Tech, Blacksburg, VA 24060, USA; yingqi6@vt.edu; 2 Management Department, School of Business, The College of New Jersey, Ewing, NJ 08618, USA

**Keywords:** Internet-of-Things, intelligent manufacturing, Li-ion battery, supply chain, direct recycling, digitalization

## Abstract

The rapid market expansion of Li-ion batteries (LIBs) leads to concerns over the appropriate disposal of hazardous battery waste and the sustainability in the supply of critical materials for LIB production. Technologies and strategies to extend the life of LIBs and reuse the materials have long been sought. Direct recycling is a more effective recycling approach than existing ones with respect to cost, energy consumption, and emissions. This approach has become increasingly more feasible due to digitalization and the adoption of the Internet-of-Things (IoT). To address the question of how IoT could enhance direct recycling of LIBs, we first highlight the importance of direct recycling in tackling the challenges in the supply chain of LIB and discuss the characteristics and application of IoT technologies, which could enhance direct recycling. Finally, we share our perspective on a paradigm where IoT could be integrated into the direct recycling process of LIBs to enhance the efficiency, intelligence, and effectiveness of the recycling process.

## 1. Introduction

The demand for Li-ion batteries (LIBs) has been growing rapidly with the expansion of the electric vehicles (EVs) sector. By 2026, the demand for LIBs is estimated to exceed 700 GWh, which would be more than six times what were produced in 2016 [1] and implies a potential market size of USD 90 billion [2]. In addition, 140 million EVs are expected to be in service by 2030 worldwide [3] and their LIBs will reach the end-of-life (EOL) in about 8–10 years [4]. It implies 340,000 metric tons of EV battery waste by 2040 which poses significant threats to the environment [5]. Specifically, hydrogen fluoride (HF) and other harmful gases would be released when the electrolyte of EOL LIBs is exposed to the air or water [6]. Meanwhile, the soil and underground water would be contaminated by heavy metals in EOL LIBs (Co, Ni, Fe and Mn) if the batteries are disposed of improperly. Moreover, Li plated or deposited in anode side shows strong reactivity and may cause explosions or fire accidents [7,8]. Meanwhile, the fast-growing demand for LIBs results in the supply shortage and the skyrocketing price of raw materials. The price of lithium and cobalt increased three times and four times respectively between 2016 and 2018 [9], which posed significant supply chain challenges to LIB production. The recycling of EOL LIBs is essential in addressing both the environmental and supply chain challenges [10,11,12,13,14]. It could reduce the reliance on the mining of raw materials in LIB manufacturing and minimize the negative environmental consequences of EOL LIBs, which enables a circular economy for LIBs.

Closed-loop recycling of LIBs could save up to 51.3% on raw materials used and 70% on energy consumption and CO_2_ emissions in LIB manufacturing [15]. The prevailing LIB recycling processes focus more on LIBs with high cobalt concentration (e.g., LIBs for portable electronics) due to the high value of cobalt. However, they may find it challenging to process EV batteries in an economically viable manner, as those batteries often contain much less cobalt. Direct recycling is believed to be a promising technological path to tackle the challenge. Life-cycle analysis indicates that direct recycling can substantially reduce the cost, energy consumption as well as emissions (e.g., CO_2_, SO_x_) in contrast to the pyrometallurgy or hydrometallurgy recycling methods [16,17,18,19,20], which suggests that it may be a more economically viable and environment-friendly recycling solution for EV batteries [11]. More importantly, the research found that direct recycling generates materials (e.g., lithium metal oxide electrode) with comparable properties to that of virgin materials [21]. Nevertheless, the barrier to the wide adoption of direct recycling lies in the fact that the ongoing development of the technology remains at the lab scale and would require digitization and intelligent technologies to make it more scalable and deployable.

Internet-of-Things (IoT) refers to a system or group of physical objects (e.g., human beings or machines) which are connected over a network and hence are able to exchange data with each other remotely and in real-time [22]. Such technology could significantly benefit industrial processes, such as battery manufacturing and recycling, of which the complicated procedures require close and continuous monitoring and control. For instance, IoT technologies could help evaluate field variables like temperature, humidity, gas evolution, and liquid viscosity in the battery recycling process [23]. Meanwhile, they enable ground floor automation when the real-time data is applied to machine learning algorithms to enable prediction, classification and decision planning with minimum or no human intervention [22]. Furthermore, IoT technologies coupled with Blockchain technologies enable reliable and secure human interfaces and valuable data analytics [24]. All of these could address the key challenges in scaling up the direct recycling process and generating electrode materials with reliable quality.

In this paper, we introduce the challenges in the supply chain of LIBs and the impact of LIB recycling. In particular, we compare the direct recycling process with the current recycling methods to highlight the benefits and challenges of direct recycling. We further discuss the characteristics and applications of IoT technologies to explore how they may enhance specific direct recycling processes. We then propose how the architecture of IoT can be integrated with direct recycling to make future recycling of spent batteries more effective. Our proposed Circular Supply Chain (CSC) framework for EOL LIB provides a more intelligent and effective solution to enable sustainable critical materials supply for LIB production at a large scale.

## 2. Challenges in the Material Supply Chain of LIBs

Table 1 provides a summary of weight, cost and supply information about primary materials used in LIBs. As the table illustrates, critical materials in LIBs, such as cobalt and lithium, are only available from a few countries, which poses significant threats to the stability of the LIB supply chain. Cobalt is widely used in the cathode active materials of batteries in EVs and consumer electronics [25]. It is known that over 60% of global cobalt production is from the Democratic Republic of the Congo (DRC), which has a long history of civil war, political instability and infrastructure problems [26]. It resulted in the unstable cobalt supply, which forced battery manufacturers to focus on developing nickel-rich and cobalt-free cathode materials like LiNi_x_Mn_y_Co_z_O_2_ (NMC), LiNi_x_Co_y_Al_z_O_2_ (NCA) and LiFePO_4_ (LFP) for EV LIBs to alleviate the supply risks [27]. However, cobalt is still an indispensable element for consumer electronics LIBs, which predominantly use LiCoO_2_ (LCO) cathode chemistry [28]. Sun et al., assessed the material supply risk of the LIB materials with an index that integrates a Herfindahl–Hirschman Index (HHI) of market concentration and Worldwide Governance Indicator (WGI) and found that the supply of cobalt is at high risk [29]. What adds to the risk is that cobalt is primarily manufactured from the by-product or co-product of other metals. Around 38% of cobalt was the by-product of nickel production, and 60% was mined from the by-product of copper [30]. The production and price of cobalt hence also fluctuate with the production of nickel and copper [31]. Stabilizing the supply of cobalt is hence an important issue for LIB production.

Lithium element has been traditionally used for the productions of lubricating greases, glasses and ceramics [32]. Its market is undergoing structural change due to the growing demand from consumer electronics LIBs. The share of lithium used in rechargeable batteries expanded from 29% in 2013 to 65% in 2019 [33]. Lithium carbonate, which is 99.5% Li_2_CO_3_, makes up the bulk of the lithium sold for electric vehicles. Its price was relatively stable (e.g., between USD 4000 and USD 5000 per ton) between 2009 and 2016, however, skyrocketed to around USD 15,000 per ton in 2017 and USD 17,000 per ton in 2018 [14]. Based on the USGS Mineral Commodity Summaries, the worldwide reserves of lithium are 17 million tons [34] so the challenge in the supply of lithium is more about whether the pace of production can meet the burgeoning demand [35]. Lithium can be extracted through distinct processes. Major lithium mining countries like Australia and Chile extract lithium from the hard rock mining from pegmatite [36] while Argentina and the U.S. recover the lithium via evaporation from the brine of salt lakes [14]. The evaporation process requires the drilling and breaking of the shell of salt brine which needs to be elevated into evaporation ponds for experiencing 12 to 24-month solar evaporation [37]. Only 6% of Li content can be extracted to produce the pure Li_2_CO_3_ for the LIBs [27]. Meanwhile, the evaporation process is also extremely water-consuming. It uses nearly 500,000 gal water per ton of lithium production [38], which may destroy the rock bed and may adversely affect the local supply of water for other purposes. The worldwide lithium production increased from 35,000 tons in 2016 to 95,000 tons in 2018, in which the market share of Australia, which adopt the hard rock mining approach, grew from 40% to 62% [39]. Therefore, to meet the explosive demand of lithium, lithium production more often adopts the hard rock mining approach and the cost is bound to rise compared with that from a brine evaporation process. Furthermore, the geographical concentration of lithium mining is becoming higher. Therefore, it is imperative to develop alternative supply of lithium.

Other metals (e.g., Mn and Ni) are increasingly used in the cathode active material to replace cobalt and thereby reduce the overall material cost of batteries [40]. However, it may result in safety concerns and other issues. For instance, nickel, which is much cheaper than cobalt, can provide high energy and power density, however, it may cause the cat-ion mixing with Li-ions due to the similar radius [41].

There are non-metal materials that may face supply chain challenges as well. For instance, graphite is widely used in the anode of LIBs [42]. While the material is generally more available, only flake graphite of high purity can be used in manufacturing LIBs, which constrains the sources of its supply. Moreover, as Table 1 shows, more than 60% of the production of graphite is based in a single country (e.g., China), which adds geopolitical risk to the supply of graphite.

**Figure 1:**
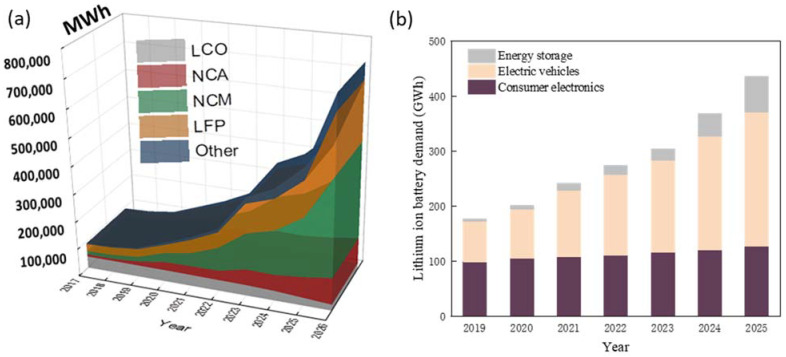
(**a**) The market of LIBs in different cathode chemistry from 2017 to 2026; (**b**) the demand of LIBs for different applications [1].

Figure 1 shows that the battery market is expected to grow rapidly due to the increasing demand for LIBs from EVs and the energy storage market. The nickel-rich cathode active materials (e.g., NCM or NCA) are expected to be more widely used while the share of LCO is estimated to decline. As mentioned, the adoption of nickel-rich cathode materials may alleviate the supply chain challenges for LIBs to some extent, however, it has inherent technical challenges and is still far from resolving the problem. Other strategies need to be developed in order to more effectively manage the supply chain risk and the cost of LIB materials.

## 3. Direct Recycling of the LIBs and Its Impact on Supply Chain

**Figure 2:**
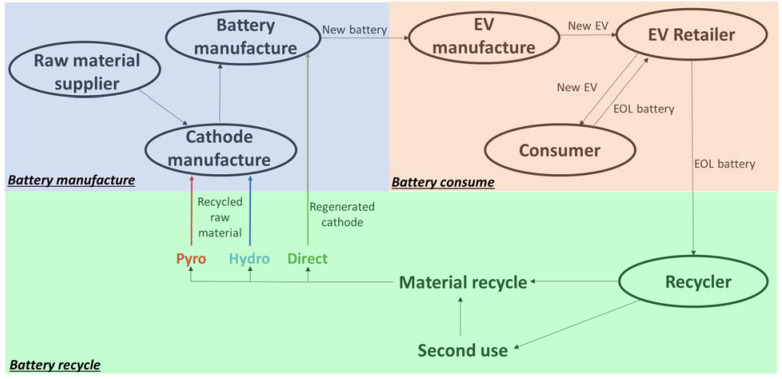
The circular supply chain of the battery industry including battery manufacturing, consuming and recycling.

Life-cycle analysis indicates that recycling end-of-life batteries could provide materials for battery manufacturing purposes and hence might be an effective solution to manage the supply chain risk in LIB production [5]. Specifically, innovation in LIB supply chain could allow materials and LIBs to flow efficiently between suppliers and customers. One example is Reverse Supply Chain Management (R-SCM), which is developed based on circular economy principles to closed-loop systems that allow battery manufacturers to receive the recovered materials from the LIBs they produced [44]. Figure 2 illustrates the circular supply chain of the battery industry. It is suggested that recycling of EOL LIBs could increase the local supply of battery materials, stabilize the material prices, reduce energy consumption and emissions in battery production, and thereby lower down the supply chain risk and the cost in battery production.

A LIB usually contains four key components: cathode, anode, separator, and electrolyte. The cathode is viewed as most critical as it contributes to a larger portion of cell cost than other components and plays a bigger role in shaping the performance of a LIB [45]. The current recycling efforts also focus more on recovering valuable metals, such as cobalt and lithium, in the cathode. The existing LIB recycling technologies generally fall into three categories: pyrometallurgy, hydrometallurgy and direct recycling process. The pyrometallurgical process (red line in Figure 2) simply dumps them into a smelter without pretreatment. It can process solid waste, ores, and any other types of batteries simultaneously. However, the pyrometallurgical process suffers from a number of drawbacks. First, the process has high energy consumption and high operating cost, which makes the recovery of lower-value materials, such as lithium and aluminum, not economically viable. It hence results in a low material recovery rate. Second, further refinement of valuable metals like cobalt, nickel, and copper through leaching or solvent extraction methods are needed before they could be reused, which makes it lengthy and more complicated to close the loop for battery manufacturers [46].

The hydrometallurgical process (blue line in Figure 2) with mechanical pretreatment and leaching process to dissolve the metal solids can recover lithium and other metals from LIBs as well. It involves the use of aqueous reagents to leach the desired metals from electrode materials. It has been proved by previous studies that acids combined with reducing agents can dissolve almost all transition metal oxides into solution. For strong inorganic acids like HCl [47,48], H_2_SO_4_ [49,50,51], and HNO_3_ [52,53], a high recovery rate can be achieved by excessive acid use. Hydrogen peroxide (H_2_O_2_) was added to accelerate the leaching reaction with less acid utilized [54,55]. Organic acids which are more environmentally friendly have also been validated to be effective for leaching transition metal materials under mild experiment conditions [56,57]. Furthermore, alkali leaching has been studied recently. Ammonia-based systems can form stable metal ammonia complexes and a fortissimo alkali such as sodium hydroxide can dissolve the cathode current collector to extract the active material [58]. The metal ions could be obtained through precipitation or solvent extraction methods to be reclaimed as salts for battery raw material [59,60]. It is believed that the hydrometallurgical process is more energy and cost-saving than pyrometallurgy in processing batteries and has a higher recovery rate of the metals in batteries [61]. However, it suffers from the drawback of generating a large volume of solvents waste, which is pollutive and costly to neutralize [62].

**Figure 3:**
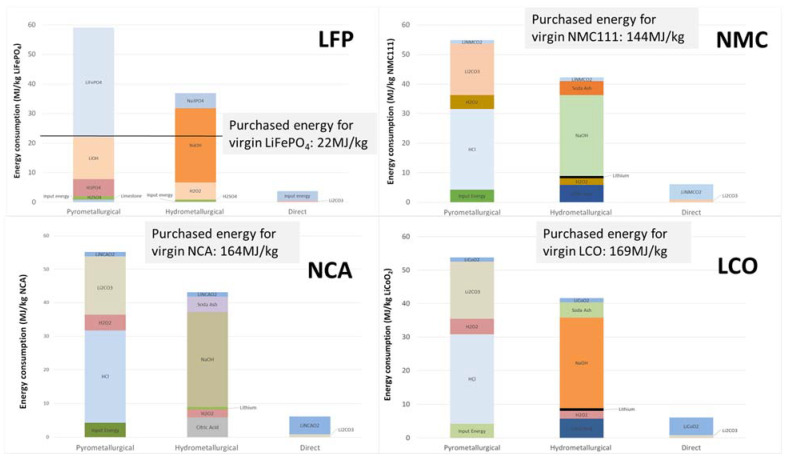
The energy consumption comparison between pyro, hydro, and direct recycling technologies for different cathode chemistry.

Direct recycling (green line in Figure 2) is to recover, recondition and reintroduce the EOL cathode or anode materials into the supply chain for manufacturing with minimum processing [11,46,63,64]. In theory, everything inside LIBs can be recycled through the direct recycling process including the electrolyte, separator, aluminum, and graphite [63]. The spent LIBs are discharged and shredded to extract the electrolyte. Cathode and anode materials are separated out for their respective regeneration processes. As the main reason for the degradation of the cathode material is the loss of lithium ions, the direct recycling process often uses re-lithiation to restore the electrochemical performance of the cathode [64]. Direct recycling is found to be the most energy-efficient and cost-effective option among all three types of recycling technologies [65]. We have compared the energy consumption, yield value, and unit cost of our direct recycling technology with incumbent pyrometallurgical and hydrometallurgical recycling technologies. As shown in Figure 3, the direct recycling of several typical LIB cathode materials (LFP, NMC111, NCA for EVs and LCO for consumer electronics) has the lowest energy consumption among the three proposed recycling approaches. In addition, the structure and morphology of the battery components are maintained through physical and chemical separations, which yield resalable high-value materials without additional processing. More importantly, as Figure 2 shows, direct recycling involves fewer steps from EOL LIBs to reusable materials, which makes it a more efficient closed-loop solution for battery manufacturers.

While extensive research has been conducted on the ways to improve yield and purity of outputs from conventional recycling processes (e.g., pyrometallurgy and hydrometallurgy), it is clear that such improvement could not fix the inherent drawback of those methods, which is mainly determined by the recovery of cobalt for their economic viability. As mentioned earlier, the industry is trending towards low-cobalt batteries for EVs, of which the recycling requires more economic and less cobalt-sensitive options, such as direct recycling.

With that being said, direct recycling still faces technical challenges, which need to be resolved to make the process a technically scalable and economically viable recycling option. One paramount challenge is the need to pre-sort batteries into a more refined battery waste stream and pre-treat them (e.g., disassembly) to enable effective processing. Direct recycling yields battery-grade material that is highly sensitive to impurity. Therefore, all procedures need to be well designed to avoid cross-contamination. Nevertheless, real-life LIBs differ in their structure, size and arrangement mode with various designs of LIB from different manufacturers [66]. Within the cells, the chemical composition of cathode active materials also varies among manufacturers and continues to evolve. Moreover, such chemical composition information is not available from labels or packages of batteries. Therefore, it is not effective for traditional manual sorting to address these problems. Meanwhile, the use of binder adhesives, sealing methods, and fixtures in current LIBs are not designed to enable easy disassembly and may result in contamination of outputs from the recycling process. Hence, it is necessary to closely monitor the experimental conditions and achieve a high quality of outputs.

## 4. The Development and Application of the Internet-of-Things

### 4.1. Overview of the IoT

The ‘Internet-of-Things’ is a manifestation of the digitalization trend firms increasingly embrace and extends the Web and Internet into the physical world through devices, sensors and their data collection and processing capabilities. By connecting physical entities through digital signals, it enables real-time or timely interaction between industrial applications and needs from customers [67]. While lacking a universal definition, IoT is generally viewed as “an open and comprehensive network of intelligent objects that have the capacity to auto organize, share information, data and resources, reacting and acting in the face of circumstance and transformation in the environment” [68]. There are four essential layers in the IoT network. The sensing layer, which can integrate the ‘things’ or existing hardware like Ratio-frequency identification (RFID) tags, actuators, sensors. The networking part takes charge of the data transmitting. The service layer is to combine applications with services using the middleware. The interface layer manages the communication between users and the system [69].

For instance, to monitor the industrial process, several connected sensor devices are often used to collect local information, such as moisture, distance, and speed (Wireless Sensor Networks, or WSN). They could also be used to control parameters like liquid flow, pressure, movement and noise in the industrial process. The data collected will then be uploaded to receivers like RFID tags through the network [70] and the middleware can be used to enable communication between sensor devices and tags or actuators. The process could be further facilitated by cloud computing, which enables resource sharing among users with access to one single platform. In such a way, large volume real-time data can be processed to allow efficient decision-making and problem-solving. [71]. Furthermore, IoT systems could facilitate interaction between humans and devices, which may eventually allow humans to transfer the idea they have in mind to machines easily. In sum, IoT technologies allow devices and humans to work more effectively together through enabling the real-time collection, transmission, and analysis of data in the industrial process and could serve as the basis for more effective decision making and optimizing the industrial process.

**Figure 4:**
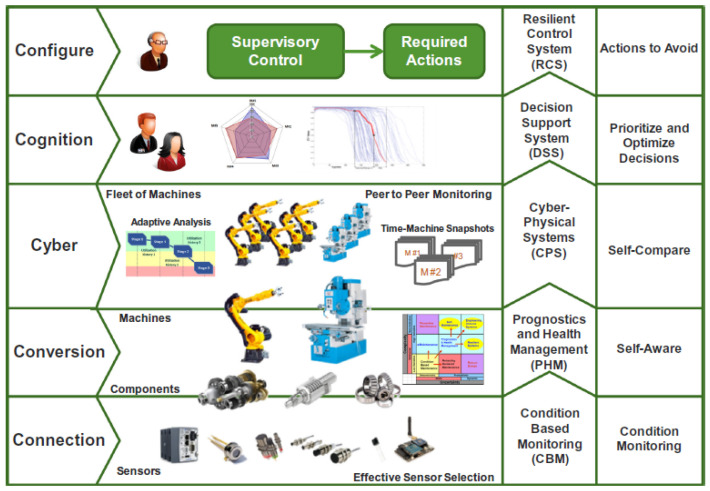
The architecture of the CPS manufacturing system. Reprinted with permission from [73]. Copyright 2015 Elsevier.

An example of the application of IoT in industrial manufacturing is cyber-physical system (CPS), which combines cloud-based manufacturing with analog hardware or digital hardware and has been investigated by several researchers [72]. The design of this system is scalable and flexible for different applications, which makes it an important architecture for intelligent manufacturing. Lee et al. [73] present a 5C architecture for CPS in Industry 4.0 manufacturing systems and provide a viable and practical guideline for implementing CPS in manufacturing for better product quality and system reliability. The applications and techniques associated with each level of the 5C architecture are shown in Figure 4. The connection level includes sensors and other devices used for acquiring data, which are then transmitted at the data-to-information conversion level and cyber level. The knowledge of the monitoring system will be eventually generated at the cognition level and be used to control physical machines at the configure level.

### 4.2. The Application of IoT in Industry

Prior studies have examined various applications of IoT. For instance, IoT can be used in industrial production [74] to help evaluate field variables through real-time sensing to realize optimal parameter conditions in manufacturing. IoT can also be used in environmental monitoring of pollution index such as humidity, temperature and noise to make it compatible with local policies [75,76,77]. Verdouw et al. [71] suggest that IoT can be used to virtualize the supply chain and enable customers to track their merchandise through the virtual control of the supply chain. IoT can provide capabilities in monitoring, inventory management, and product tracking to make supply chain management (SCM) more effective. Sandip et al. conduct a data-driven analysis on SCM particularly for the automobile battery industry in India [78]. It is suggested that IoT enables firms to be more intelligent through increasing visibility and transparency in SCM, enhancing customer experiences, and empowering businesses with an agile supply chain [79].

IoT would be especially beneficial for the direct recycling process of LIB in RSC, which has a more delicate process design and requires more refined collection and treatment strategies for different battery waste streams. Specifically, IoT would enable the faster collection of battery waste, lower processing costs and higher output value in the recovery process of batteries. It would also enable more effective communication and information sharing between manufacturers and customers [80]. IoT would also allow the process to track, monitor and control the distribution, quality and cost of the recovered materials, which is difficult to do with the traditional supply chain system. In sum, IoT is well suited to address the uncertainties and challenges of RSC [44].

### 4.3. Blockchain and Its Integration with IoT (BIoT)

The adoption of an IoT system requires a high level of trust among different entities (nodes, gateways, users) in the system. It hence necessitates the integration of blockchain technologies with IoT. Blockchain technologies are characterized by decentralization, pseudonymity, fault tolerance as well as adaptability [81]. They are able to track, coordinate, carry out transactions and store information from various devices without dependence on a centralized cloud.

Smart contracts in blockchain networks served as the agent to execute the transactions in a secure and immutable manner [82]. The integration of blockchain with IoT is particularly beneficial in the energy and CleanTech sectors. For instance, Aitzhan and Svetinovic introduced a cutting-edge secure transaction exchange system using blockchain for decentralized energy trading in another CPS-based, smart grids [83]. The blockchain-based IoT system has also been used to offer whole-life-cycle tracking of battery materials to ensure transparency in sources of materials, ethical supply chains, and battery health [84].

## 5. The IoT Enhanced Direct Recycling Process for LIB

### 5.1. The Application of IoT Devices in Different LIB Direct Recycling Processes

It is believed that direct recycling of LIBs involves interdependent processes, which co-determine the quality of final outputs. The direct recycling of EOL batteries starts from battery collection and classification. The batteries are then deactivated and disassembled through physical pre-treatment, which is followed by a separation process to extract the electrode materials from the batteries. After that, material regeneration processes, such as solid-state sintering, re-lithiation, and hydrothermal, are conducted to restore the performance of the electrode materials [62]. Drawing on the direct recycling process described above, we explore the possibility of applying IoT in each stage of the direct recycling process and propose a paradigm for an IoT enhanced direct recycling process of LIBs:

(1). Collection and classification

Direct recycling of LIBs enables a closed-loop recycling solution for battery manufacturers as it could return the battery grade electrode materials that the manufacturers used in production. Such an approach has several merits: it ensures stability in material supply and consistency in material quality. It also makes the adoption easier as recycled materials may have similar physical characteristics such as particle size compared to virgin materials that are currently used. However, the challenge lies in how to obtain or create LIB waste streams that are consistent in their cathode materials used. Effective battery classification and sorting during or after battery collection hence become highly important for a direct recycling process.

**Figure 5:**
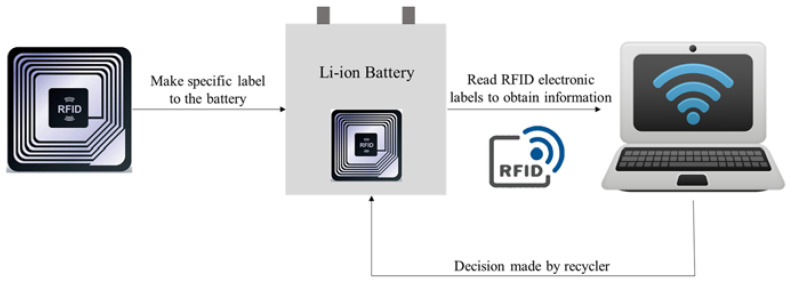
Illustration of RFID labeled LIB cell for information tracking.

We suggest that battery collection and classification should be easier if batteries are designed for such a purpose. For instance, RFID is one of the major communication standards to achieve short-range seamless connectivity [85]. RFID tags could be placed on the batteries so that the batteries could be tracked and when being transported across the RFID-enabled conveyor belts. The information, such as the chemical components or dates of manufacturing, in the attached tags can also be updated in each stage of production. Such a process can be done on a battery cell or a battery module. As noted, direct recycling requires the inputs (e.g., EOL LIBs) to be consistent in their cathode chemistry to yield regenerated cathode material of high purity. The use of RFID would allow real-time traceability and effective classification, which make the subsequent processes of direct recycling smoother and simpler. Figure 5 below illustrates the concept of the RFID labeled LIB cell.

Blockchain technology is also integrated with IoT to store battery information and build an efficient battery evaluation and recovery platform [86]. Sun et al. introduce an Ethereum blockchain-based rich-thin-clients IoT solution for electric vehicle battery swapping [87]. A smart contract is developed for the battery swapping system, which can manage both static and dynamic information. Static information refers to the information which is fixed once the batteries are formed and unchangeable, such as manufacturer, production date, and manufacturing price. Dynamic information refers to information about manufacturers, state of charge (SOC), power capacity and refueling history, which will be updated over time.

For EOL batteries without RFID tags, an intelligent recognition method could be effective for classification and diagnosis purposes. Convolutional neural network (CNN) is a deep learning technique that has produced promising results in solving problems with image classification and could automatically discover the representation needed for the task at hand [88]. The multi-layered neural networks can extract the features of a pattern and are well-known for their robustness to small inputs variations, minimal pre-processing, and low requirement for specific feature extractor choice [89]. CNN can be used to classify batteries based on their types and is appropriate for large-scale applications since it can automatically optimize its hyperparameters with the auspice of the deep learning models. CNN could also be used to evaluate the condition of LIB electrodes and automatically detect microstructural defects based on light microscopy images [90]. Hence, it can be used to separate defective batteries from good ones and the data generated in the process could enable opportunities to further optimize battery recycling with IoT technologies.

(2). Disassembly

The disassembly of spent LIBs also poses challenges to direct recycling. Specifically, the possible cross-contamination between different battery components (e.g., cathode or anode) in the disassembly process could compromise the performance of the recycled materials. In particular, as the end product of direct recycling is battery-grade materials, it is critical to precisely separate out the electrode material and purify the segregated materials.

Another concern with battery disassembly is the safety issue (e.g., risk of fire or explosion) in the process. Deactivation or stabilization techniques before disassembly are required to prevent thermal runaway. It is expected that discharging to 2.5 V open circuit voltage (OCV) or lower in LCO or NMC—graphite cell will release more than 98% energy [91]. The deactivation process can be achieved by discharging in brine or other heat-resistant conductors. Ideally, the residual energy of the spent batteries could be collected and sold as discharging to 0 V or less may result in contamination in end products due to the dissolution of Cu into electrolyte [92,93]. The level of energy and voltage can be monitored in real-time using IoT technology to ensure appropriate discharging. IoT could also enable in-process stabilization using engineer controls such as aqueous spray or inert gas atmosphere during comminution or shredding to limit risks [94,95].

**Figure 6:**
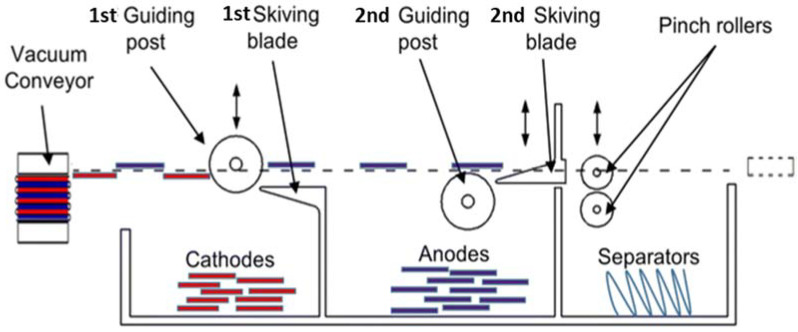
The prototype for automatic sorting of LIB pouch cell [96].

IoT technology is also essential for automated battery disassembly. Currently, the disassembly process is manual at the lab scale, in which workers are exposed to toxic substances in the spent batteries and the risk of battery explosion [63]. The manual disassembly process is also generally costly, time-consuming and unstable in its outputs. Alternative disassembly methods such as crushing introduce a tremendous amount of impurities like copper, aluminum into the outputs of disassembly, which makes the downstream recycling process much more difficult. Therefore, a more automated and refined disassembly process is needed to ensure high accuracy, high processing speed and high purity of outputs, which is particularly important for the direct recycling process. We have demonstrated the prototype machinery that can dismantle mimic 2 Ah LIB pouch cells and automatically sort various cell components [96]. Figure 6 illustrates the automated sorting device that can effectively separate the cathode sheets, anode sheets and the separator. It also suggests that IoT technologies are needed to make the disassembly process more automatic and intelligent. For instance, sensors like thermal imagers can be employed to monitor the whole process, especially the temperature and bespoke sensors are needed for the perception of product information. Computer vision and tactile force sensing are required to process the complicated scenarios with dynamic interface of force. The data generated from these sensor devices would then be analyzed in real-time to continuously optimize the process.

(3). Separation

The purpose of separation in the direct recycling process is to obtain the desired material with high purity and quality. Specifically, it separates the electrode powder material from other components such as current collector, electrolyte as well as pouch cases. The design of such a process is often evaluated based on its efficiency, cost, emission and impurity.

Previous literature introduces several separation methods including physical processes like heat treatment and solvent wash assisted with ultra-sonication [2,97]. Organic reagents like N-methylpyrrolidone (NMP) or N,N-dimethylformamide (DMF) are used to dissolve the binder and weaken the adhesion. Notably, mild working condition is preferred as it would result in less aluminum debris being generated and introduced into black mass material. It is reported that excessive aluminum impurity would deteriorate the performance of cathode material [98]. Meanwhile, chemical leaching has been employed to dissolve the aluminum current collector [99]. The feedstocks from the shredding or comminution processes need to be consistent in their size so that they could be further separated by other physical processes such as flotation separation [100].

Multiple sensors can be used to monitor the separation process to provide real-time data and improve the efficiency of separation. For example, the increase of the Solid/Liquid (S/L) ratio in the black mass suspension could deteriorate the efficiency of separation. Proper sensors for the viscosity or transparency can be employed to monitor the S/L ratio in the separation process. Furthermore, computer vision can be used to analyze the real-time images of the current collector after the separation process to control quality. Future studies could explore these applications to further optimize the direct recycling process for LIBs.

(4). Regeneration

**Figure 7:**
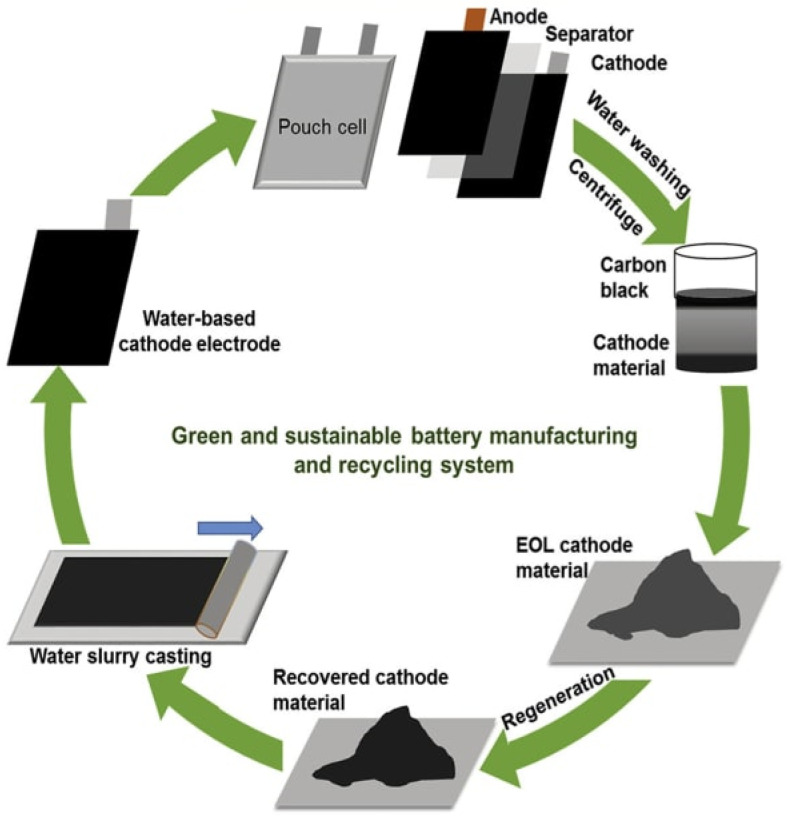
Schematic of the battery green manufacturing and direct recycling process to form the circular supply chain [12].

As illustrated in Figure 7, the regeneration process is an essential step in the direct recycling of LIB. Specifically, the process of regeneration repairs the electrode materials and restores their chemical composition as well as crystal structures, resulting in the comparable performance of electrode material, including specific capacity, cycling stability, and rate capability. Great efforts have been made to directly regenerate cathode materials. Normally high temperature or dynamic support is required to realize the stoichiometry recovery of electrode material. The regenerated powder materials are then rejuvenated in their crystal structure, morphology, element ratio as well as electrochemical properties.

IoT technologies could be used in the regeneration process to monitor several parameters, which are critical to the quality of the final products. For instance, sensors could be used to monitor and collect real-time data on factors like temperature, amount of lithium added and time-lapsed, etc. Advanced algorithms could be applied to understand the correlation between process setting and material characterization to optimize the performance of the final outputs. For example, Jiang et al., trained a model for water addition by exploiting the real-time and historical performing data among the sintering process. The combination of offline deep supervised learning and online unsupervised learning algorithm is proposed to achieve the effective prediction and control of the moisture [101].

Solid-state sintering has been used to regenerate cathode materials from EOL LCO, LFP and NMC LIBs [102,103]. The process is similar to the synthesis process in cathode powder production and can heal the material effectively. Prior studies used a machine learning-based prediction model to investigate the optimal ratio of raw material solutions using a combination of X-ray diffraction (XRD) patterns and the experimentally derived characteristics [104], which provide a helpful use case of a machine learning algorithm in the physical synthesis process. Beyond solid-state sintering, re-lithiation can also be achieved through more complicated methods, such as hydrothermal [105], electrochemical [64,106], and chemical processes [107]. The benefits of integrating IoT in direct recycling increase once the methods/processes become more complicated (e.g., involving more steps or more parameters to be monitored) as real-data processing and analysis become more essential tools for process optimization.

### 5.2. The Application of Artificial Intelligence and Machine Learning in Direct Recycling of LIBs

Machine learning and artificial intelligence technologies have gained great attention in recent years to enhance the value creation of IoT technologies. In particular, machine learning, particularly deep learning, algorithms could unlock the value of the abundance of real-time data collected by IoT sensors in enabling more automated, faster and more effective decisions.

As noted, the direct recycling of LIBs enables a closed-loop solution for LIB manufacturers. Therefore, a more intelligent information-sharing platform for LIB recyclers and manufacturers is essential in supporting this closed-loop solution. Celia et al. [108] presented a multi-stage framework containing collection, assessment, dismantling, material recovery and shipping of the EV batteries in which an IoT system powered by machine learning algorithms is integrated to bring intelligence to the ‘Things’ like containers, pallets, and trucks. In particular, data on inventory and products is collected through the sensors embedded on trucks, pallets, containers and battery modules as well as the RFID tags placed on the cover of batteries and is then shared in the system to enhance its transparency.

Moreover, more intelligent IoT technologies are critical in enabling cleaner battery waste streams for recycling. As noted, the effectiveness of direct recycling is contingent on the consistency in the feedstock of battery wastes. Smart IoT technologies powered by machine learning could allow firms to sort spent batteries by their electrode materials based on data collected by the sensor about the batteries. At the same time, such data could allow smart segregation of different batteries and intelligent classification of used batteries into streams for re-manufacture, reuse and recycling. Such an analysis could be done based on the data collected on cell component chemistries, the state of charge and state of health of the cells and could lead to more refined sorting and much less component contamination, which is essential for direct recycling. In sum, IoT technologies could create larger value for the recycling process once being combined with machine learning algorithms, which could convert the data they collected into effective decisions.

### 5.3. Proposed Paradigm

**Figure 8:**
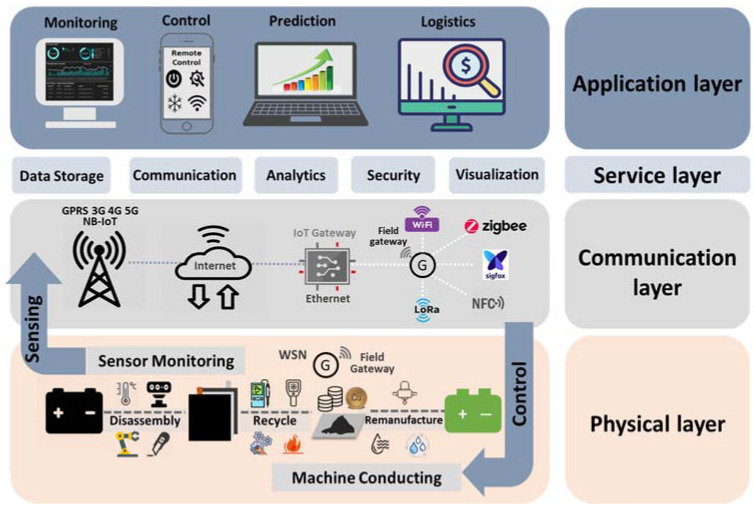
Proposed paradigm for IoT enhanced direct recycling of LIBs application [109].

**Figure 9:**
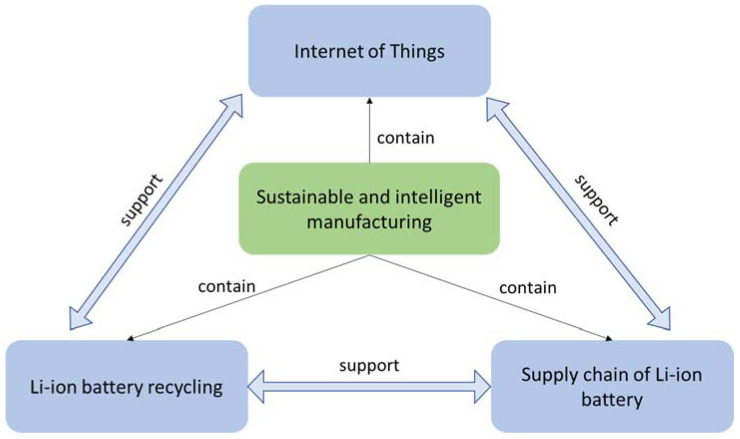
Three aspects of the sustainable and intelligent eco-system of LIBs [110].

We propose an IoT integrated paradigm for direct recycling of spent LIBs, which is illustrated in Figure 8. In particular, there are four layers in the proposed paradigm, namely application layer, communication layer, service layer and physical layer. The physical layer includes the actual production line of the direct recycling process, in which multiple IoT sensors or devices are embedded to collect important data on batteries themselves and the recycling process. Above the physical layer is the communication layer, which helps with transmitting data from the physical layer to the service layer. Specifically, the key objective of the communication layer achieves the information transmission between physical world and cyber world through IoT gateways based either on mobile networks or Ethernet. It contains field gateways acting as interfaces between IoT gateways and transceivers using ZigBee, WiFi, Sigfox, Bluetooth or LoRA. The service layer provides tools with which data received from the communication layer could be ingested and utilized. The top application layer leverages the tools in the service layer and the data collected at the physical layer to monitor, control and keep optimizing the process of LIB recycling. The application layer also enables decisions, such as how to sort batteries, how to separate battery components and how to regenerate and restore the performance of electrode materials effectively. The expected outcomes from this IoT enhanced direct recycling process of LIBs are faster and more refined sorting of batteries, higher purity of outputs from the separation process as well as high-performing electrode materials recovered from spent batteries. As shown in Figure 9, we believe IoT, direct recycling and the supply chain of LIB are interconnected, mutually reinforcing and all essential in enabling a more sustainable and intelligent ecosystem of LIBs.

## 6. Conclusions

In this paper, we first review challenges in the LIB supply chain and highlight the importance of developing a direct recycling process of LIBs to mitigate those challenges. The characteristics of direct recycling compared with conventional recycling technologies are illustrated. We then discuss the characteristics and applications of IoT technologies to explore the feasibility of using IoT to enhance different processes in the direct recycling of LIBs. We present a paradigm of IoT integrated direct recycling system as a solution to tackle the challenges in the direct recycling process of LIBs. 

## Figures and Tables

**Table 1:** Summary of 60 kWh NMC532/graphite battery materials [34,43].

Electrode	Element	Weight Percent (kg/kWh)	Cost Percent (USD/kWh)	Mine Production and Supply Information Worldwide
Cathode	Cobalt	0.22	40.77	14,800 tons: 60% Congo (Kinshasa)
	Nickel	0.55		2,400,000 tons: 25% Indonesia, 15% Philippines
	Manganese	0.31		18,900 tons: 31% South Africa, 18% Australia
	Lithium	0.13		95,000 tons: 62% Australia, 18% Chile
	Aluminum	0.22	0.03	63,600 tons: 56% China
Anode	Copper	0.46	0.08	20,400 tons: 29% Chile, 12% Peru
	Graphite	1.1	20.43	1,120,000 tons: 62% China

## Data Availability

Data sharing not applicable.
